# Spontaneous coronary artery dissection in cutis laxa

**DOI:** 10.1259/bjrcr.20210248

**Published:** 2022-11-01

**Authors:** Pia Frances Pemberton Charters, Daniel Barnaby McKenzie, Jonathan Carl Luis Rodrigues, William Wynn Loughborough

**Affiliations:** 1 Royal United Hospitals Bath NHS Foundation Trust, Bath, UK; 2 Bristol Heart Institute, Bristol, UK; 3 Department for Health, University of Bath, Bath, UK

## Abstract

We describe the case of a 21-year-old female with Cutis Laxa presenting with an acute coronary syndrome. A CT coronary angiogram (CTCA) diagnosed spontaneous coronary artery dissection (SCAD) of the right coronary artery, which was successfully managed with medical therapy. Cutis Laxa is a rare connective-tissue disorder in which the skin becomes inelastic. Lax, redundant skin hangs in folds give a prematurely aged appearance and several forms of the disease have been described. Although certain cardiovascular complications are recognised in Cutis Laxa, to our knowledge SCAD has not been previously described.

SCAD is an uncommon cause of acute coronary syndrome and sudden cardiac death. The condition particularly affects young females, those with connective tissue diseases, arteriopathies, pregnant females, contraceptive use and cocaine use. Atherosclerotic risk factors are seldom reported. The condition is underdiagnosed as symptoms may not generate a high index of clinical suspicion in this demographic. Diagnosis is traditionally made on invasive coronary angiogram although the procedure carries risks in SCAD and non-invasive CTCA should be considered in appropriately selected patient cohorts or as an adjunctive measure to assess for extracoronary vascular abnormalities. Our patient was diagnosed on CTCA, avoiding the need for invasive catheter angiogram.

## Clinical presentation

A 21-year-old female presented to the Emergency Department with a 12 h history of sharp, central chest pain. The pain initially awoke her from sleep and worsened throughout the day to a maximum severity of 9/10. Pain was associated with transient shortness of breath on minimal exertion and three episodes of witnessed syncope. Two weeks prior to admission, she experienced a non-specific viral illness which caused generalised malaise and was associated with a 30 min episode of similar chest pain that resolved spontaneously.^1–3^

She had a relevant past medical history of Cutis Laxa diagnosed at age 17. The diagnosis was made clinically, based on childhood photos, personal and familial phenotype. Cardiovascular risk factors include a family history of maternal diabetes and hypertension. She was an active smoker with a one pack year history and took no regular medication. On examination, her heart rate was 52 beats per minute with a blood pressure of 101/60  mmHg and oxygen saturation of 98% on air. Clinical examination was unremarkable. Her ECG showed sinus rhythm with no ischaemic changes. D-dimer was normal while troponin T was mildly raised at 17 ng l^−1^ (<14  ng l^−1^) with interval rise to 33 ng l^−1^ at 2 h.

## Differential diagnosis

Chest pain and syncope with a troponin rise should prompt investigation and management of an acute coronary syndrome (ACS). In the context of a known connective tissue disorder, coronary artery dissection should be considered. Differentials include aortic dissection, coronary artery aneurysm, coronary artery spasm/arrhythmia and myopericarditis.

## Investigation/imaging findings

### Primary investigation

On admission, she was initially investigated with an ECG gated CT angiogram aorta (CTA), which did not demonstrate an aortic dissection or central pulmonary embolus (heart rate 70–72 beats per minute). Bedside transthoracic echocardiography was performed, which showed good left and right ventricular systolic function without regional wall motion abnormality.

### Diagnostic investigation

Her chest pain resolved over 2 h and there were no subsequent ECG changes, therefore she was admitted for observation with a cardiac monitor. Repeat troponin on day 2 was significantly elevated at 731 ng l^−1^ (asymptomatic). At this point, the possibility of a SCAD was raised. Diagnostic imaging options included invasive coronary angiography (ICA), optical coherence tomography (OCT) and CT coronary angiography (CTCA)—see discussion section for further detail on these imaging modalities. The cardiology and radiology teams concluded that CTCA was the optimal test for this patient as it is a non-invasive test with less risks than invasive options. Additionally, if complete occlusion is unlikely due to the lack of ongoing pain or ECG changes (as in this case), current practice is to manage SCAD conservatively, negating the need for invasive intervention.

**Figure 1.**
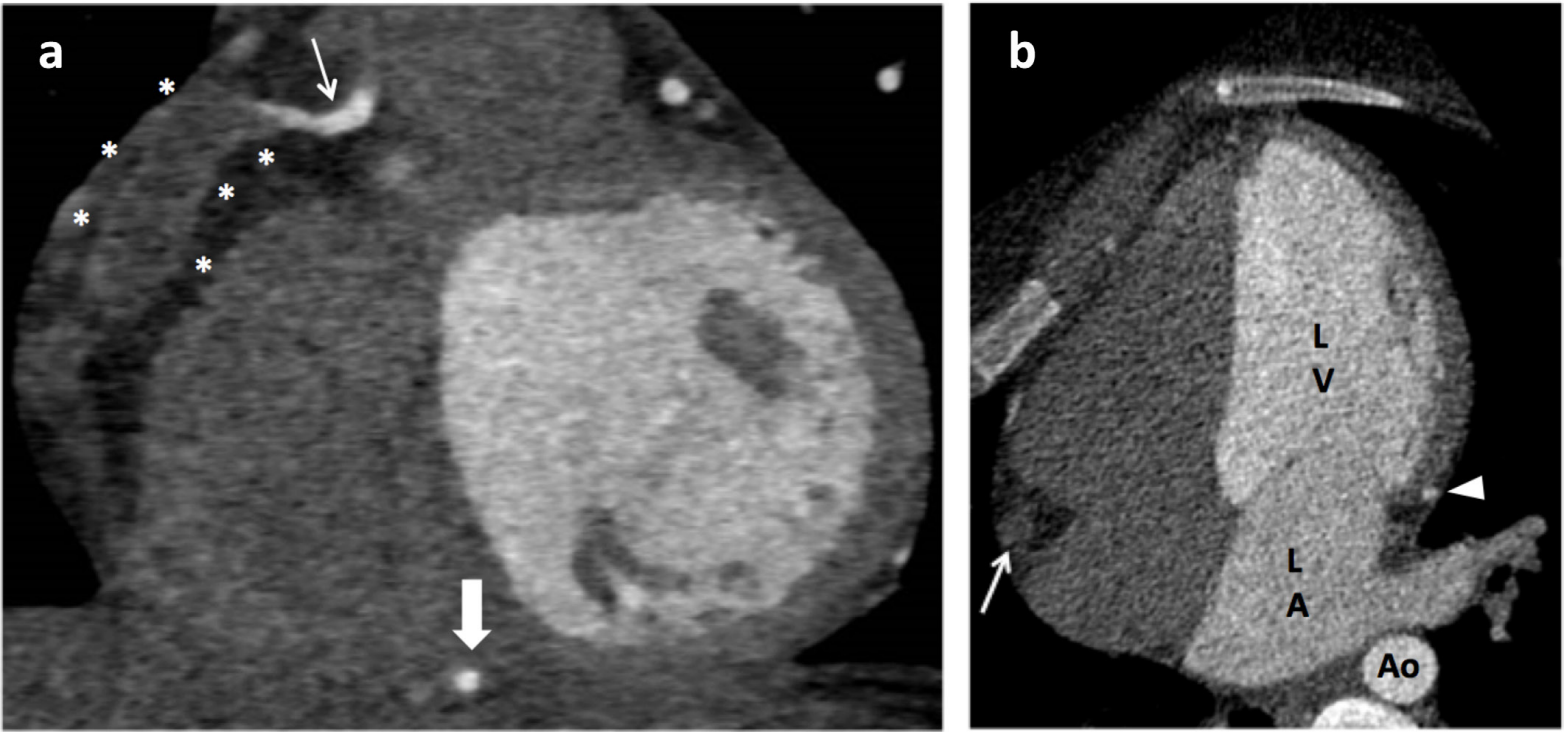
*CT coronary angiogram demonstrating a right coronary artery dissection.* 1 .a—coronal slice demonstrating the right coronary artery within the atrioventricular groove. The proximal right coronary artery is normal calibre and opacified with contrast suggesting patency (thin arrow). The patent vessel then tapers into a non-opacified dissected vessel expanded with thrombus (stars). There is distal refill of the PDA branch (thick arrow) via collaterals. 1.b—four chamber view demonstrating the right coronary artery expanded with thrombus (arrow). The left circumflex coronary artery is normal calibre and opacified with contrast suggesting patency (arrow head). Ao, aorta; LA, left atrium; LV, left ventricle.

**Figure 2.**
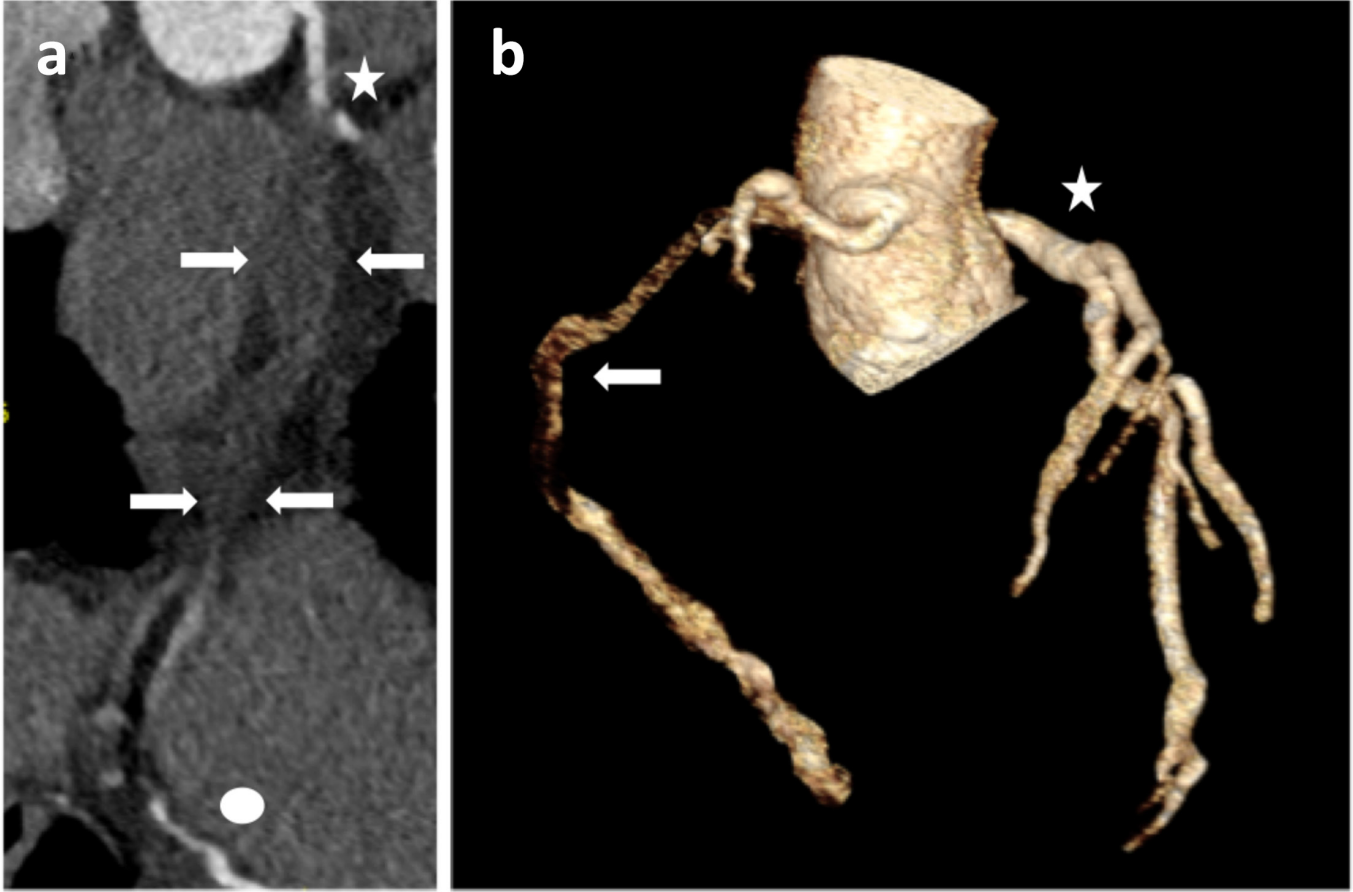
2 .a—Curved mutiplanar reconstruction of the RCA. Patent proximal RCA (star). Mid RCA is dissected and expanded with thrombosis with no intraluminal contrast (arrows). Distal vessel (PDA) is opacified from filling via collaterals (circle). 2.b—Volume rendered three dimensional reconstruction of the coronary arteries. Dissected RCA (arrow) has poor definition (due to lack of intraluminal enhancement) compared to well defined and normally opacified left sided coronary arteries (star). RCA, right coronary artery.

A FLASH prospective diastolic CTCA was performed on Dual Source CT scanner (SOMATOM^®^ Drive, Siemens Healthineers, Erlangen, Germany). Standard acquisition parameters were used; automated tube current modulation with 145 reference mAs and automatic dose modulation, 120 kVp tube voltage with automated kV modulation, 1.2 pitch, 0.5 s rotation time and 512 × 512 acquisition matrix. Prospective end-diastolic ECG gating was performed without the need for intravenous beta-blockade (heart rate 58–60 bpm). The intravenous contrast protocol was 80mls of Omnipaque 350 at 4ml/s with bolus tracking. This study demonstrated occlusion of the right coronary artery (RCA) extending from the acute marginal branch to the posterior descending artery (Figures 1 and 2). The patent proximal RCA tapers into a non-opacified and expanded segment of vessel which was hyperdense on pre-contrast imaging, presumed thrombus. Spontaneous coronary artery dissection was considered to be the most likely diagnosis, with thrombus either filling the false lumen and/or intramural haematoma. The distal vessel was reconstituted by collaterals. Another differential would be fissuring of a non-calcified plaque in the context of ACS. However, the radiological features were more in keeping with SCAD, there was no atherosclerotic disease present and SCAD was clinically felt to be more likely in a female of this age with no risk factors for ACS. In retrospect, the initial CTA demonstrated occlusion and expansion of the right coronary artery, although findings were more subtle than on the dedicated CTCA given the comparatively higher heart rate.

## Treatment

She was managed medically for a non-ST elevated myocardial infarction (NSTEMI) with acetylsalicylic acid (ASA) monotherapy and β blockade. Her pain had settled, she had no ECG changes and a repeat echocardiogram demonstrated good left and right ventricular systolic function. Given the resolution of pain, a non-invasive initial diagnostic approach was favoured.

## Outcome/follow-up

She made an excellent recovery without symptom recurrence or ECG change and was discharged after 4 days. She remained systemically well 6 months after discharge with normal repeat echocardiography. She will be reviewed by the SCAD team in Leicester.

## Discussion

Cutis Laxa is both genetically and clinically heterogeneous. Several forms of the disease have been described which include at least eight specific gene mutations, autosomal recessive and autosomal dominant patterns, an extremely rare X-linked inheritance and an acquired form.^1^ Traditionally the diagnosis was made clinically following identification of the classic phenotype, as with our patient, although contemporary diagnosis has moved towards molecular subtyping given the discrete clinical courses. Recognised radiological findings include potentially life-threatening pulmonary emphysema, bronchiectasis, hernias of the diaphragm and abdominal wall, genitourinary prolapse and diverticulosis of the bladder and gastrointestinal system.^3^ Cardiovascular abnormalities include arterial tortuosity, aortic aneurysms and aortic dissection.

SCAD is an epicardial coronary artery dissection that is non-atherosclerotic, non-traumatic and non-iatrogenic. Two theories of how SCAD develops have been described. The first theory proposes that the primary pathological event is a disruption in the intima, allowing blood to enter the wall from the lumen.^2^ The second theory proposes that the primary event is spontaneous haemorrhage in the vasa vasorum of the vessel wall.^4^ Regardless of the initial mechanism, SCAD is characterised by the spontaneous formation of haematoma in the coronary wall (specifically the outer third of the media), leading to progressive narrowing of the true lumen and potential myocardial ischaemia and infarction. In this case report, we postulate a similar series of events; dissection of the intima caused pain at presentation but without vessel occlusion and only mild troponin rise. As the coronary wall expanded with haematoma, the vessel lumen became occluded causing myocardial ischaemia and a subsequent troponin rise.

SCAD is reported to represent 1–4% of all ACS cases,^2^ although as an underdiagnosed condition the true prevalence is likely greater. The condition typically affects young females and given the paucity of atherosclerotic risk factors, even classic ACS symptoms may not generate a high index of clinical suspicion.^2^ Other risk factors include connective tissue disease (*e.g.* Ehlers-Danlos and Marfans), arteriopathies (*e.g.* fibromuscular dysplasia (FMD)), arteriopathies, pregnancy, contraceptive use and cocaine use.^2^

The three main diagnostic imaging techniques for SCAD are ICA, intravascular imaging and/or CTCA. ICA has been the traditional diagnostic technique with the highest sensitivity and the added benefit for intraprocedural primary percutaneous intervention (PCI) where necessary. Performing ICA in suspected SCAD has the potential risk of propagating the dissection further when wiring the vessel. Addition limitations include increased cost and centre-dependent accessibility. Intravascular imaging in the form of ultrasound and OCT is complimentary to ICA and can potentially detect intimal tears, false lumen and intramural haematoma.^2^ However in the context of suspected SCAD, there is the concern that the high pressure contrast injection used for OCT may extend dissections.

CTCA is emerging as an efficient tool for coronary assessment. It is a non-invasive, low cost and quick study which is readily available in most UK centres. Compared with conventional angiography, CTCA is limited by an intrinsically lower spatial resolution and cannot reliably assess the luminal diameter and walls of small vessels (<2.5 mm).^2^ SCAD tends to affect the distal coronary arteries which are both of a smaller caliber and more likely to be affected by artefact on CTCA, thus ICA remains the gold-standard test.^2^ However, CTCA as a non-invasive test is a reasonable first line option in appropriately selected patients suspected of SCAD. There is a paucity of prospective data on how CTCA compares with ICA in diagnosing suspected SCAD and sensitivity and specificity for diagnosis have not yet been defined.^4^ CTCA findings in SCAD include lack of atherosclerotic plaque, tapered luminal stenosis, abrupt luminal stenosis, luminal occlusion, intramural haematoma, dissection flap and perivascular epicardial fat stranding.^5^ Additionally, patients with SCAD have a high prevalence of coronary artery tortuosity and myocardial bridging which can be readily identified with CTCA and may define a high risk phenotype.^5^ The role of CTCA is being explored and currently adds value as a non-invasive follow-up study^2^ particularly of larger proximal arteries or as an adjunctive measure to assess for extracoronary vascular abnormalities.

In this case, following CTCA the patient’s pain had settled, there were no ECG changes and an echocardiogram demonstrated good left and right ventricular systolic function. The CTCA indicates that preserved function was likely due to retrograde filling from left coronary artery collaterals, which preserved inferior wall perfusion. It was felt that proceeding to invasive angiography with its associated risks would be unlikely to add value.

Traditionally, management of SCAD reflected the management of coronary artery atherosclerotic disease, although the conventional approach has moved away from PCI towards medical therapy. No randomised control trial has been conducted to support the use of PCI and there is concern that PCI may increase the likelihood of vascular complications in SCAD.^6,7^ Complications described in the PCI treatment of SCAD include late stent thrombosis, propagating the intramural haematoma and extending the dissection thus further compromising perfusion.^8^

Some centres recommend screening all patients with SCAD for arteriopathies, particularly FMD.^2^ There is currently no consensus on which screening modality to use, with ultrasound, CT, MRI and invasive angiography performed according to accessibility and centre preference. Research into effectiveness of screening is limited. The Mayo Clinic are currently creating a genomic DNA and plasma biobank of SCAD patients with view to identifying causative mutations.^9^ This will inform future research into the management and prognosis of SCAD and in turn the value of screening.

### Informed consent statement

Written informed consent was obtained from the patient for publication of this case report, including accompanying images.

## Learning points

- For acute medical doctors: in the context of a known connective tissue disorder, chest pain and syncope with a troponin rise should prompt consideration of SCAD. Missed diagnoses of SCAD are often secondary to low clinical suspicion of ACS in young females. Increasing clinician awareness of the condition will allow early diagnosis and optimum management.
- For radiologists: when assessing gated CT thoracic imaging for chest pain, it is essential to review the coronaries (particularly in young females), as there may be features suggestive of SCAD. Adequate rate control improves coronary assessment.
- For cardiologists: consider CTCA as the initial diagnostic tool in suspected cases of SCAD.
